# Effects of a Web-Based Computer-Tailored Game to Reduce Binge Drinking Among Dutch Adolescents: A Cluster Randomized Controlled Trial

**DOI:** 10.2196/jmir.4708

**Published:** 2016-02-03

**Authors:** Astrid Jander, Rik Crutzen, Liesbeth Mercken, Math Candel, Hein de Vries

**Affiliations:** ^1^ School for Public Health and Primary Care CAPHRI Department of Health Promotion Maastricht University Maastricht Netherlands; ^2^ School for Public Health and Primary Care CAPHRI Department of Methodology and Statistics Maastricht University Maastricht Netherlands

**Keywords:** adolescents, alcohol drinking, binge drinking, cluster randomized controlled trial, serious games, computer tailoring

## Abstract

**Background:**

Binge drinking among Dutch adolescents is among the highest in Europe. Few interventions so far have focused on adolescents aged 15 to 19 years. Because binge drinking increases significantly during those years, it is important to develop binge drinking prevention programs for this group. Web-based computer-tailored interventions can be an effective tool for reducing this behavior in adolescents. Embedding the computer-tailored intervention in a serious game may make it more attractive to adolescents.

**Objective:**

The aim was to assess whether a Web-based computer-tailored intervention is effective in reducing binge drinking in Dutch adolescents aged 15 to 19 years. Secondary outcomes were reduction in excessive drinking and overall consumption during the previous week. Personal characteristics associated with program adherence were also investigated.

**Methods:**

A cluster randomized controlled trial was conducted among 34 Dutch schools. Each school was randomized into either an experimental (n=1622) or a control (n=1027) condition. Baseline assessment took place in January and February 2014. At baseline, demographic variables and alcohol use were assessed. Follow-up assessment of alcohol use took place 4 months later (May and June 2014). After the baseline assessment, participants in the experimental condition started with the intervention consisting of a game about alcohol in which computer-tailored feedback regarding motivational characteristics was embedded. Participants in the control condition only received the baseline questionnaire. Both groups received the 4-month follow-up questionnaire. Effects of the intervention were assessed using logistic regression mixed models analyses for binge and excessive drinking and linear regression mixed models analyses for weekly consumption. Factors associated with intervention adherence in the experimental condition were explored by means of a linear regression model.

**Results:**

In total, 2649 adolescents participated in the baseline assessment. At follow-up, 824 (31.11%) adolescents returned. The intervention was effective in reducing binge drinking among adolescents aged 15 years (*P*=.03) and those aged 16 years when they participated in at least 2 intervention sessions (*P*=.04). Interaction effects between excessive drinking and educational level (*P*=.08) and between weekly consumption and age (*P*=.09) were found; however, in-depth analyses revealed no significant subgroup effects for both interaction effects. Additional analyses revealed that prolonged use of the intervention was associated with stronger effects for binge drinking. Yet, overall adherence to the intervention was low. Analyses revealed that being Protestant, female, younger, a nonbinge drinker, and having a higher educational background were associated with adherence.

**Conclusions:**

The intervention was effective for adolescents aged 15 and 16 years concerning binge drinking. Prevention messages may be more effective for those at the start of their drinking career, whereas other methods may be needed for those with a longer history of alcohol consumption. Despite using game elements, intervention completion was low.

**Trial Registration:**

Dutch Trial Register: NTR4048; http://www.trialregister.nl/trialreg/admin/rctview.asp?TC=4048 (Archived by WebCite® at http://www.webcitation.org/6eSJD3FiY)

## Introduction

Alcohol use in adolescents, especially dangerous drinking practices such as binge drinking (drinking ≥4/≥5 glasses of alcohol in one occasion for a girl/boy) and excessive drinking (drinking ≥10 glasses of alcohol on one occasion) [1], are associated with detrimental long- and short-term consequences. Alcohol use is the cause of 26% of all deaths in males and 10% of all deaths in females between the ages of 15 and 29 years in Europe [2]. Also, short-term consequences, such as physical fighting and injuries [3], dating violence, unintended pregnancies, and illicit drug use [4-6], are severe and influential experiences for adolescents. Particularly, the influence of alcohol on the developing brain can lead to serious brain damage, cognitive deficits, and learning disabilities [7-9].

In the Netherlands in 2011, 57.4% of adolescents aged 16 years and 61.9% aged 17 to 18 years reported that they had engaged in binge drinking at least once in the previous 30 days [10], with significantly more boys reporting binge drinking (70.5%) than girls (53.1%). Compared to other European countries, this is relatively high [11]. Moreover, a Dutch survey from 2013 shows that of the 16-year-old adolescents who reported drinking alcohol in the previous month, 79.9% also reported binge drinking [12]. However, these data were collected when adolescents were allowed to buy alcoholic beverages with an alcohol content of less than 15% when they turned 16 years of age (as of January 1, 2014, the legal age to buy any alcoholic beverages was increased to 18 years). Still, these adolescents grew up in an environment where drinking from the age of 16 was acceptable and relatively common [10], as the previously mentioned surveys show. Hence, targeting adolescents’ motivation to decrease alcohol use and binge drinking is important.

Changing alcohol use in adolescents could be achieved with the help of Web-based computer-tailored interventions [13]. In the Netherlands, 97% of the population aged between 12 and 65 years has access to the Internet [14]. Differences in access between Dutch social classes range from 92% for lower-educated adolescents to 99% for higher-educated adolescents. Thus, Web-based computer-tailored health interventions have the potential to reach many people from various social classes and ages. These interventions provide the opportunity to tailor health messages to individual characteristics of the recipient (eg, demographics and motivational variables), which result in highly personalized and relevant messages that are more likely to attract attention [15]. Computer-tailored interventions have been shown repeatedly to effectively change various health behaviors and their determinants [16-18], although their effect sizes are generally small to medium. However, Web-based computer-tailored interventions suffer from high dropout rates (eg, one study reported a 37% dropout rate after 6 months [13] and another a 72% dropout rate after 12 months [19]; the mean adherence rate to Web-based health interventions is 50% [20]) with at least 2 negative consequences. First, high dropout rates during the intervention result in nonexposure to the intervention leading to reduced public health impact. Second, high dropout rates also result in less power to reveal intervention effects at follow-up because people who drop out during the intervention are also not likely to participate in a follow-up assessment [21].

This is related to a general difficulty engaging adolescents in health interventions [22,23]. Yet, using serious games (ie, games with the goal to educate people rather than merely entertain them) [24,25] to change health behaviors could lead to more attraction and participation [26,27], increased knowledge, and changed attitudes and behavior [24,25]. Consequently, our study employed a serious game as a method to provide computer-tailored feedback.

Furthermore, parents still play an important role in preventing adolescents from drinking too much alcohol. Studies have shown that setting clear rules [28,29] and good quality communication with the child about alcohol [30,31] has positive effects on the child in terms of less alcohol consumption. However, another study suggests that communication between parents and adolescents is virtually absent when Dutch adolescents turn age 16 [32]. Therefore, we also provided computer-tailored feedback to parents concerning how to set clear and consistent rules with regard to alcohol use and how to communicate clearly with their child about alcohol.

The aim of this study was to test the effectiveness of a Web-based computer-tailored intervention after 4 months to reduce binge drinking (ie, drinking ≥4/≥5 glasses of alcohol for a girl/boy on one occasion) in Dutch adolescents aged 15 to 19 years; as a secondary outcome, we also assessed the effects of the intervention on excessive alcohol use (ie, drinking ≥10 glasses of alcohol on one occasion) and alcohol consumption during the previous week (ie, the sum of glasses consumed during the previous week). Furthermore, we assessed differential intervention effects concerning age, gender, and educational level.

## Methods

### Study Design

As of January 1, 2014, the legal age to purchase alcohol in the Netherlands increased from 16 to 18 years [33], which had some implications for our study design. Originally, the baseline assessment was planned for October 2013 and the follow-up assessment for April 2014. However, to avoid the law change (and its potential impact on drinking behavior) taking place between the baseline and follow-up assessments, we delayed the baseline assessment until after the law change was in effect in January 2014. Furthermore, we decided on a 4-month follow-up assessment instead of a 6-month follow-up because a 6-month follow-up assessment would have fallen in the summer vacation period.

We conducted a cluster randomized controlled trial (RCT) (trial registration number: NTR4048), randomizing Dutch schools of either lower secondary education and lower vocational training or higher secondary education into an experimental and a control condition. The experimental condition received the online intervention in the form of a game that contained computer-tailored feedback. The control condition only filled in the online baseline questionnaire. Both groups were given an online follow-up assessment after 4 months, responding to the same questionnaire as used in the baseline assessment. The study took place in the Netherlands between January and June 2014.

### Participants and Procedure

Adolescents were recruited in schools. Information letters addressed to teachers and coordinators of the highest grades at secondary education schools (grades 4-6) and at vocational training schools were sent via postal mail. These information letters informed the teachers about the intervention and provided contact details and the address of the study website, so schools could obtain more information and subscribe to the study. All eligible schools in the Netherlands (approximately 600 schools) received an invitation. If schools did not respond, they were called 2 to 3 weeks later. Schools were randomly assigned to either the experimental or control condition after their consent to participate in the study. Schools were not blind to their condition because experimental schools had to plan 3 lessons for the intervention (one lesson for the baseline assessment and first game session, a second lesson for the second and third game session, and a third lesson for the 4-month follow-up assessment) and control schools had to plan 2 lessons (the baseline assessment and the 4-month follow-up).

Approximately 3 weeks before the intervention started, teachers were provided with a letter containing more information about the procedure, privacy, and confidentiality. All adolescents in the classes were provided with a letter on the day of the intervention to avoid them starting the intervention prematurely. The letter informed the adolescents that all their answers in this study would never be shared with teachers, parents, or any other third person; would be used for research purposes only; would be analyzed anonymously; and that they could end participation at any point in time. Adolescents were also made aware that, at the end of the study, they would participate in a lottery for 300 gift vouchers worth €25 each. Furthermore, adolescents were provided with a letter for their parents. In this letter, the parents were informed that their child participated in an online alcohol intervention at school and the parents were invited to visit a separate website specifically for parents, where they could take part in the parental component of the intervention. When starting with the intervention, teachers asked the adolescents to visit the study website and create an account. They were routed to the according condition (either control or experimental) based on the school they attended. Before starting with the baseline questionnaire, all adolescents had to check a box on the first page of the website that contained informed consent information confirming their informed consent. If they did not wish to participate or refused to provide informed consent, they could check an “I do not wish to participate in this study” box; they were thanked and could then close the intervention website (see Multimedia Appendix 1 for the CONSORT eHealth checklist filled out for this study).

### Inclusion Criteria

Our main target group was adolescents aged 16 to 18 years. Because we were recruiting the adolescents in schools, we also included younger (15 years) and older (19 years) adolescents because they are often in the same class. Schools had to provide the adolescents with individual access to a computer with Internet connection.

### Intervention

The idea of a game instead of a purely text-based computer-tailored intervention was first brought up by adolescents during focus group interviews [32]. During the development of the intervention, all materials and questions concerning the game (eg, its name, screenshots and characters of the game, realistic scenarios after drinking too much alcohol, realistic advice for adolescents who are trying to drink less in tempting situations, layout and design of the first version of the game) were presented to a Facebook panel. This panel consisted of a convenience sample of 24 adolescents aged 16 to 18 years, who provided us with feedback on those materials. The feedback was used to adapt the game to match the desires of the target group as closely as possible. After the development was completed, the game was pilot-tested at 5 schools to test the feasibility of the recruitment strategy, the design, and the content of the intervention. In total, 481 adolescents played the first game session and provided us with feedback about appreciation, comprehension, attractiveness, and level of personalization of the game. They were also asked about what they liked and disliked about the game. Based on this pilot, we shortened the game and the feedback messages were shortened and rewritten by a professional writer to make them more appealing to our target group. Originally, only the first game session was offered in the school and the adolescents were asked to continue with the game at home. After reviewing the feedback and the pilot data, we decided to make some changes to the design and to offer all 3 game sessions within the school setting.

**Figure 1:**
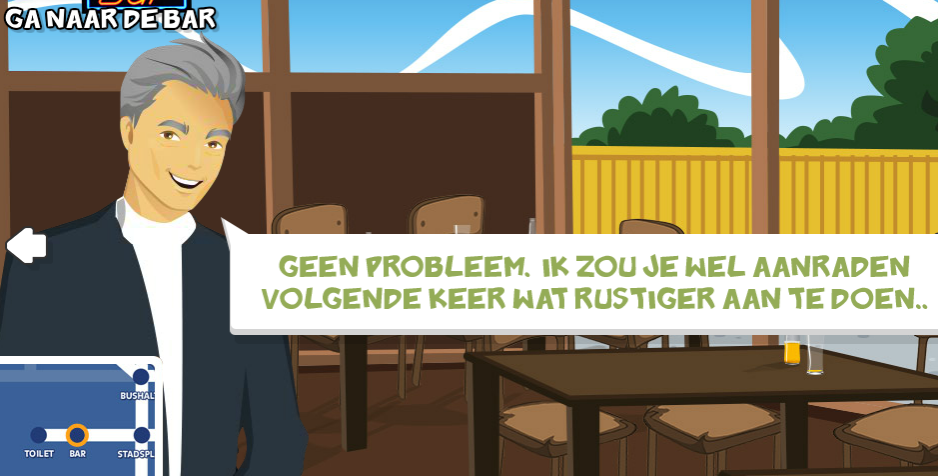
Screenshot example from the game (In the bar: “No problem. I would suggest you keep it down a little next time...”).

The intervention, Alcohol Alert, consisted of an online baseline questionnaire, after which the adolescents played 3 sessions of the game “What happened?!”. In these game sessions, the adolescent wakes up after a night of partying and does not remember what happened the night before. The goal of this 2-dimensional game was to find out what happened. Each of the game sessions depicted one of the most common drinking situations for adolescents (ie, drinking at home, drinking in a bar, drinking at a party). The sequence of the 3 game sessions was tailored and dependent on how many glasses of alcohol the adolescent indicated to typically drink in each of these situations. The adolescent started with the drinking situation in which he or she indicated drinking the most alcohol. Thus, if the adolescent indicated typically drinking 3 glasses at home, 5 glasses at a party, and 6 glasses in a bar, he or she would start with the bar scenario first, followed by the party scenario, and finally the home scenario. Each session started in the bedroom where the adolescent wakes up. The adolescent quickly discovers that something is wrong (eg, the wallet is missing in one session). The adolescent then navigates through different places in the game and talks to people he or she meets and gets clues about what happened the previous night (Figure 1).

**Figure 2:**
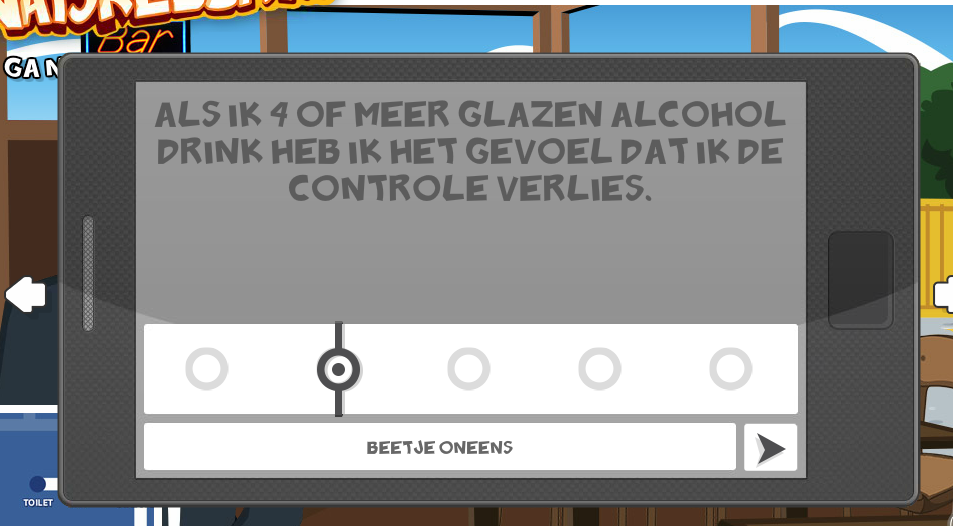
Screenshot example of the in-game cell phone (Question: “If I drink 4 glasses of alcohol or more I feel like I am losing control”).

During the game sessions, the adolescent received questions and feedback on an in-game cell phone (Figure 2). These questions and feedback were based on the I-Change Model [34], an integrated model based on theories such as the Attitude-Social Influence-Self-Efficacy Model [35], the Theory of Reasoned Action [36], the Theory of Planned Behavior [37], Social Cognitive Theory [38], the Health Believe Model [39], the Precaution Adoption Model [40], and the Transtheoretical Model [41]. The I-Change Model attempts to explain motivational and behavioral change and has been successfully used to design and evaluate health interventions previously [19,42,43]. The questions and computer-tailored feedback were based on the relevant concepts of the I-Change Model (ie, attitude, modeling, social norm, perceived pressure, and self-efficacy). Within the game, this was operationalized by presenting the in-game cell phone twice during every game session. During the first presentation of the first game session, the adolescents were asked questions about their attitude toward binge drinking and received immediate feedback to try to shift their attitude about binge drinking to be less positive. The first time the in-game cell phone was presented in the second scenario, questions about modeling of alcohol use and binge drinking were asked (ie, who in their family and friends engaged in binge drinking), and feedback was provided to help the adolescents to choose the right role models. The first time the cell phone was presented in the third scenario, questions concerning social norms (ie, if parents and friends approved of drinking) and perceived pressure (ie, whether the adolescents perceived pressure to binge drink from family or friends) were posed and the feedback messages tried to encourage adolescents to resist that pressure. The second time the cell phone was presented during each scenario, questions about situation-specific self-efficacy were posed (eg, in the bar scenario adolescents were asked how difficult it is for them not to binge drink in a bar). Feedback was provided to enhance self-efficacy and the adolescent was provided with action plans that he or she could use in that particular situation. We decided on this sequence based on the Ø pattern [44], which describes that people shift toward behavior change through first developing a favorable attitude, experiencing positive social influences, and finally developing high self-efficacy toward the behavior.

The content and methods used in the feedback messages varied depending on the message, but usually the answer of the respondent was repeated to enhance self-monitoring, correct assumptions were confirmed with positive feedback, and wrong assumptions were corrected with new information. All messages had a personal tone to show sympathy and to enhance commitment [45]. For example, the attitude questions assessed the pros (eg, “binge drinking helps me relax and connect easily with other people”) and cons (eg, “binge drinking makes me feel like I am losing control”) of binge drinking. The adolescent immediately received feedback on his or her overall attitude and for every pro and con specifically. In these feedback messages, the focus was on providing the participant with general (eg, “alcohol inhibits your brain’s natural inhibition system”) and personal (eg, “you might say or do things that you regret later”) consequences of alcohol to change attitude toward binge drinking to a more negative one. For more information about the content of the feedback messages, we refer to our study protocol [46]. Adolescents received 2 reminder emails to finish the game sessions if they did not do so at school; the first after 1 week and the second after 2 weeks. A week after the third game session, the adolescents were invited to revisit the intervention and received 2 reminder emails; the first after 1 week and the second after 2 weeks if they did not return. In this fourth session, which was not part of the game, alcohol use during the last week was assessed and the adolescents were provided with feedback about their use compared to Dutch drinking guidelines. Following this, the adolescents were asked if they had an event in the upcoming 30 days (eg, a party, wedding) where they usually drink 4 (for girls) / 5 (for boys) or more glasses of alcohol on such an occasion. If they responded positively, they were asked if they wanted to challenge themselves to drink less than they usually would. If they responded positively again, they were asked to indicate the date of the event and how many glasses they wanted to drink at most. They could then make their own action plans for how to achieve their goal or they could indicate from a list of action plans which one they would most likely follow to achieve their goal. If adolescents indicated that they had no event in the upcoming 30 days or that they did not wish to participate in the challenge, they only received advice on how action plans could help them to prevent binge drinking in the future. At the end of the fourth session, all adolescents were provided with the feedback they received during the game to boost their memory. One day before the drinking event, they were reminded by email that they accepted a challenge to drink less alcohol than they usually would for the event the next day. Two days after the event, they were invited to come back to the website to indicate if they met their goal. Reminder emails were sent after 1 and 2 weeks if they did not return. If they indicated they drank more than they had planned, they received feedback on how to do better next time and were given the opportunity to repeat the challenge. If they indicated that they had not exceeded their drinking maximum, they received congratulations and the intervention was over. For a detailed description of the development and the content of the intervention, we refer to the study protocol of this study [46].

After 4 months, the adolescents in both conditions responded to the online follow-up questionnaire in school. If they did not finish the follow-up assessment at school, they received 2 reminders to do so at 1 and 2 weeks after the official deadline for the schools.

### Parental Component

A separate component was added to the intervention to involve parents. During the development, a convenience sample of 14 parents provided us with feedback on the layout, usability, and content of the parental component. At baseline, adolescents in the experimental condition were asked to enter the email address of one of their parents. Parents then received an email inviting them to a separate website, where parents responded to a short questionnaire and could also receive computer-tailored feedback on how to set appropriate rules concerning alcohol use and how to communicate with their child about alcohol use. If the adolescent did not know the email address of the parent or did not wish to send an email to the parent, they could refuse to do so. They were informed that the letter they received for their parents contained all the information about the parental component and instructions on how parents could participate. A detailed description of the parental component can be found in the study protocol [46].

### Measures

#### Demographics

At baseline, we assessed gender (0=female, 1=male), age (in years), educational level (1=higher secondary education, 0=lower secondary education and vocational training), religion (Catholic, Protestant, Muslim, other religion, no religion), and ethnicity (Dutch, Antilles, Belgium, German, Suriname, Moroccan, Turkish, other; later dichotomized into 0=non-Dutch, 1=Dutch).

#### Binge Drinking, Excessive Drinking, and Weekly Consumption

We assessed different forms of alcohol use at baseline and at the 4-month follow-up. We assessed binge drinking, the primary outcome, with an open-ended question: “How often did you drink 4 (for girls) / 5 (for boys) or more glasses of alcohol on one occasion in the previous 30 days?” [47]. Binge drinking was later dichotomized (0=reported no binge drinking, 1=reported binge drinking). Furthermore, we assessed alcohol use in the previous week with 2 questions: “On which days during the past week did you drink alcohol?” (Monday to Sunday, I haven’t drank in the past week, I never drink any alcohol) and how many glasses of alcohol they drank on each of the drinking days if they indicated that they drank at least one day during the past week. Weekly consumption was calculated by counting the total number of glasses they drank in the past week [48]. Finally, someone was characterized as an excessive drinker if they had at least one drinking occasion of 10 or more glasses of alcohol [1] during the previous week. Weekly consumption and excessive drinking were considered as secondary outcomes [46].

#### I-Change Concepts (Attitude, Modeling, Social Norm, Perceived Pressure, Self-Efficacy)

For a description on how these concepts were assessed, we refer to the study protocol [46]. Reliability and validity information about these concepts are presented in Table 1. The eigenvalue presents an estimate of the explained variance and should be at least 1 [49]. The McDonald hierarchical omega is an estimator for factor saturation regarding the general factor; the value is a less-biased alternative to Cronbach alpha [50]. Both indexes support comprehensive assessment of questionnaire quality [51].

**Table 1:** Eigenvalues and omega of the I-Change concepts.

Scale	Eigenvalue	Omega	Cronbach alpha
Pros	3.09	.87	.90
Cons	2.55	.78	.81
Modeling of alcohol use	2.75	.68	.74
Modeling of binge drinking	2.67	.46	.72
Social norm	4.37	.83	.92
Perceived pressure	4.69	.85	.94
Self-efficacy	6.41	.81	.94

#### Adherence

Adherence was assessed by counting the number of intervention sessions (not the baseline assessment or follow-up assessment) in which the adolescent participated ranging from zero (did not participate in a single intervention session) to 5 (participated in all 5 intervention sessions).

#### Power Analyses

The primary outcome was the difference in number of binge drinking occasions in the preceding 30 days for the experimental group compared to the control group. Based on prevalence data from the time the study was designed, we aimed at reducing reported binge drinking occasions from 70% to 60% in the previous 30 days. We used the Optimal Design Plus Empirical Evidence (version 3.0) program [52]. Because adolescents were nested in schools, a cluster RCT was needed. Using a conservative approach with an estimated intraclass correlation coefficient of .02, power of .80, significance level of .05, with approximately 100 students participating per school, and considering dropout of 50% of adolescents at primary follow-up, the program indicated that 30 schools should be included. To correct for unequal numbers of students per school, we added 14% more schools [53] and aimed to include 34 schools at baseline.

#### Statistical Analyses

This study constituted a design with 3 levels: repeated measurements, nested within adolescents, nested within schools. The data were analyzed using SPSS version 20. Descriptive statistics were used to describe the characteristics of the baseline sample. Differences between the conditions in the baseline sample were assessed using *t* tests for continuous variables and chi-square tests for discrete variables. Chi-square tests and *t* tests were further used to describe differences between completers and participants who did not return to the follow-up assessment after 4 months.

To determine the effectiveness of the program, we analyzed the data with a logistic regression mixed models analysis for the outcomes binge drinking and excessive drinking and a linear regression mixed models analysis for the outcome weekly consumption. These models allow for dependencies among observations obtained for students within a school. These analysis models also allow for data missing at random, which is less strict than the requirement of data missing completely at random [54]. The variables (ie, condition, gender, age, educational level, religion, ethnicity, parental participation) as well as the interaction effects between condition and gender, age, and educational level were entered as covariates into the analyses.

The associations between potential participant characteristics (gender, age, educational level, religion, ethnicity, and binge drinking at baseline) and adherence (ie, the number of intervention sessions the participant passed through) to the intervention were analyzed using a linear regression model.

Main effects were considered significant if *P*≤.05. Interaction effects were considered significant if *P*≤.10.

### Ethics Committee Approval

This trial was reviewed by the Medical Ethics Committee of Atrium Orbis Zuyd and was classified as research that does not fall under the Medical Research Involving Human Subjects Act and needed no further approval (METC number: 12-N-104).

## Results

### Participation and Attrition

**Figure 3:**
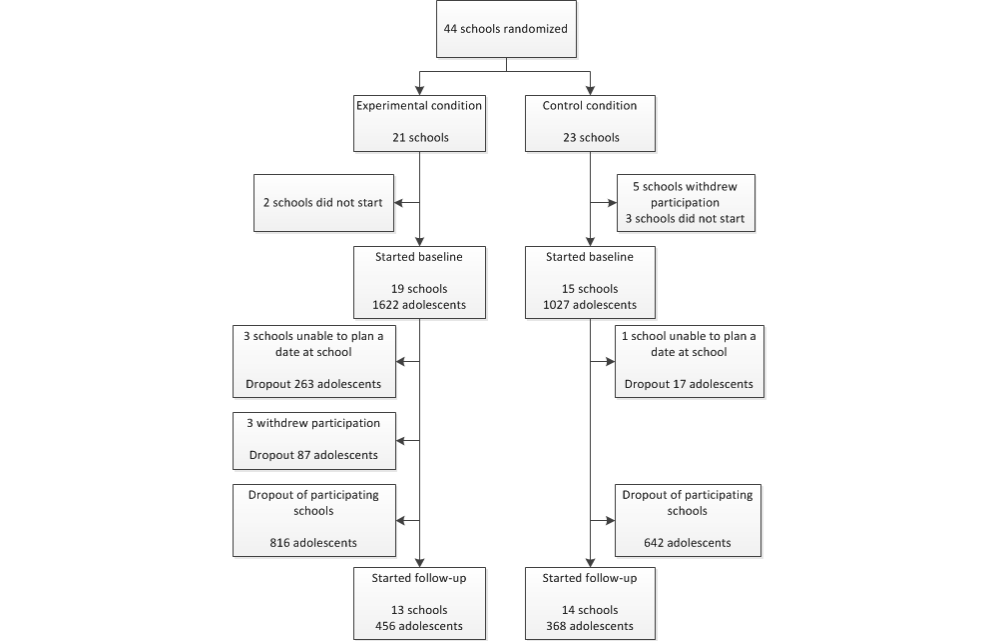
Flowchart of participation.

Figure 3 depicts a flowchart of the participating schools. In total, 44 schools were randomized into the experimental or control condition. Five schools in the control condition withdrew their participation before the baseline assessment started (2 schools of secondary higher education, 1 school of secondary lower education, 1 school of lower vocational training, and 1 school of secondary education mixed). Three schools in the control condition (all secondary higher education) and 2 schools in the experimental condition (1 lower vocational education, 1 higher secondary education) did not start with the baseline assessment and did not respond to our phone calls and emails. Most schools that dropped out before the intervention started indicated that they had logistical problems (eg, they had no computer room available to provide every adolescent with his or her own computer). Another school decided after randomization that the topic was too sensitive and they did not want to do that at school. In total, 2649 adolescents from 34 schools participated in the baseline questionnaire. The adolescents in the 2 conditions significantly differed from each other in various characteristics. Participants in the experimental condition were significantly younger, consisted of more females, had a higher educational level, more often indicated to be religious, and consisted of more participants who never drink, were less often binge and excessive drinkers, and had a lower weekly consumption than participants in the control condition (Table 2). Even though 27 schools participated in the 4-month follow-up questionnaire, only 824 of 2649 adolescents (response rate 31.11%) did so. Schools that withdrew participation at the follow-up assessment either reported trouble with finding a date due to the final exams of the classes or indicated that the adolescents were not keen to continue with the intervention and, therefore, the school decided to stop participation. Dropout analyses revealed that adolescents returning to the follow-up questionnaire were significantly younger, more often female, had a higher educational level, were more likely be religious, were more often Dutch, were less likely to be excessive drinkers, less likely to be binge drinkers, and had a lower weekly consumption (Table 3).

**Table 2:** Baseline characteristics of participants and differences between the experimental and control groups at baseline.

Variable^a^		Total N=2649	Experimental n=1622	Control n=1027	Baseline difference		*P*
					*t* (*df*)	χ^2^ (*df*)	
Age (range 15-19 years), mean SD (missing=16)		16.3 (1.2)	16.0 (1.2)	16.7 (1.2)	15.01 (2633)		<.001
**Gender, n (%) (missing=11)**						38.6 (1)	<.001
	Male	1395 (52.66)	766 (47.23)	629 (61.25)			
	Female	1243 (46.92)	847 (52.22)	396 (38.56)			
**Educational level, n (%) (missing=11)**						92.6 (1)	<.001
	High	1546 (58.36)	1056 (65.10)	490 (47.71)			
	Low	1092 (41.22)	557 (34.34)	535 (52.09)			
**Religion, n (%) (missing=17)**						33.2 (4)	<.001
	Catholic	610 (23.03)	407 (25.09)	203 (19.76)			
	Protestant	180 (6.79)	133 (8.19)	47 (4.58)			
	Muslim	165 (6.23)	82 (5.06)	83 (8.08)			
	Other	131 (4.95)	81 (4.99)	50 (4.87)			
	No religion	1546 (58.36)	907 (55.92)	639 (62.22)			
**Ethnicity, n (%)**						1.7 (1)	.19
	Dutch	2326 (87.81)	1434 (88.41)	892 (86.85)			
	Non-Dutch	323 (12.19)	188 (11.59)	135 (13.15)			
**Alcohol use, n (%)**							
	Never	710 (26.80)	491 (30.27)	219 (21.32)		25.4 (1)	<.001
	Binge drinking (missing=3)	1343 (50.70)	758 (46.73)	585 (56.96)		26.3 (1)	<.001
	Excessive drinking (missing=18)	245 (9.25)	116 (7.15)	129 (12.56)		21.5 (1)	<.001
	Weekly consumption, mean (SD) (missing=18)	4.0 (9.4)	3.4 (8.9)	5.1 (9.9)	4.57 (2631)		<.001
**Parental participation**							
	Invited by adolescent, n		199	—			
	Start, n (%)		91 (45.7)	—			
	End, n (%)		76 (83.5)	—			

^a^ Number of missing values per variable indicated as “missing.”

**Table 3:** Differences between adolescents that returned or dropped out at follow-up.

Variable^a^		Returned n=824	Dropped out n=1825	Dropout difference		*P*
				*t* (*df*)	χ^2^ (*df*)	
Age (range 15-19 years), mean SD (missing=16)		16.2 (1.2)	16.4 (1.3)	–3.87 (2633)		<.001
**Gender, n (%) (missing=11)**					20.8 (1)	<.001
	Male	381 (46.3)	1014 (55.56)			
	Female	442 (53.7)	801 (43.89)			
**Educational level, n (%) (missing=11)**					68.0 (1)	<.001
	High	579 (70.4)	967 (52.98)			
	Low	244 (29.6)	848 (46.46)			
**Religion, n (%) (missing=17)**					9.5 (4)	.049
	Catholic	208 (25.3)	402 (22.03)			
	Protestant	46 (5.6)	134 (7.34)			
	Muslim	46 (5.6)	119 (6.52)			
	Other	31 (3.8)	100 (5.48)			
	No religion	490 (59.7)	1056 (57.86)			
**Ethnicity, n (%) (missing=0)**					10.7 (1)	.001
	Dutch	749 (90.9)	1577 (86.41)			
	Non-Dutch	75 (9.1)	248 (13.58)			
**Alcohol use, n (%)**						
	Never (missing=0)	229 (27.8)	481 (26.35)		0.5 (1)	.46
	Binge drinking (missing=3)	370 (44.8)	973 (53.32)		16.7 (1)	<.001
	Excessive drinking (missing=18)	50 (6.1)	195 (10.68)		14.6 (1)	<.001
	Weekly consumption, mean (SD) (missing=18)	2.8 (6.5)	4.6 (10.4)	–5.51 (2631)		<.001

^a^ Number of missing values per variable indicated as “missing.”

### Binge Drinking

At the baseline assessment, 758 of 1622 (46.73%) adolescents in the experimental and 585 of 1027 (56.96%) adolescents in the control condition reported binge drinking in the previous 30 days. At the follow-up assessment, 194 of 456 (42.6%) adolescents in the experimental condition and 184 of 368 (50.0%) adolescents in the control condition reported binge drinking in the previous 30 days. The returning sample did not differ on baseline drinking characteristics. They did not differ on being a drinker (control: 274/368, 74.4%; experimental: 322/456, 70.6%; χ^2^_1_=1.3, *P*=.25) on binge drinking (control: 167/368, 45.4%; experimental: 203/456, 44.5%; χ^2^_1_=0.4, *P*=.83), on excessive drinking (control: 28/368, 7.6%; experimental: 22/456, 4.8%; χ^2^_1_=2.7, *P*=.10), nor on weekly consumption (control: mean 3.2, SD 6.9; experimental: mean 2.4, SD 6.1; *t*_819_=1.62, *P*=.11). There was a significant interaction effect between condition and age (*P*=.08) (Table 4). Age groups were analyzed separately using the pick-a-point approach [55] by centering the age variable for each year from ages 15 to 19. This way, the whole sample could be used to determine whether the intervention was effective for one or more of the age groups. Information about binge drinking prevalence at baseline and follow-up per age group are available in Table 4. Analyses revealed a significant effect of the intervention in 15-year-old adolescents (*P*=.03). Adolescents in the experimental group reported a significant decrease in binge drinking in the previous 30 days at 4 months after the intervention ended compared to adolescents in the control condition. Adolescents in the experimental group aged 16 years also engaged less in binge drinking after 4 months compared to the control group. This effect was not significant (OR 0.56, 95% CI 0.30-1.05, *P*=.07) (Table 5), but could be considered a small effect [56]. Furthermore, although participation of parents was very low (Table 2), when parents participated in the intervention, their participating child reported less binge drinking in the previous 30 days (*P*=.04). A higher educational level (*P*<.001), a lower age (*P*<.001), and being Protestant (*P*=.03), Muslim (*P*<.001), or a member of another religion (*P*=.03) (all analyzed in a model without interaction terms) were significant protective determinants of binge drinking (Table 5).

**Table 4:** Prevalence rates of binge drinking per age group.

Age (years)	Experimental condition, n/n (%)		Control condition, n/n (%)	
	Baseline n=1608	Follow-up n=453	Baseline n=1025	Follow-up n=368
15	222/688 (32.2)	51/180 (28.3)	54/173 (31.2)	35/105 (32.7)
16	255/479 (53.3)	71/155 (45.8)	153/303 (50.5)	57/128 (44.5)
17	146/246 (59.3)	40/64 (63.5)	200/288 (69.4)	52/76 (68.4)
18	81/112 (72.3)	11/22 (50)	111/144 (77.6)	20/26 (76.9)
19	53/83 (64.6)	19/32 (59.4)	67/117 (57.3)	20/30 (66.7)
Total	757/1608 (47.13)	192/453 (42.5)	585/1025 (57.1)	184/368 (50.1)

**Table 5:** Effects of the intervention on binge drinking, excessive drinking and weekly consumption in the complete model.

Variable^a^		Binge drinking		Excessive drinking		Weekly consumption	
		OR (95% CI)	*P*	OR (95% CI)	*P*	β (SE)	*P*
Condition (control)		0.40 (0.18-0.83)	.01	0.48 (0.18-1.25)	.13	1.82 (1.39)	.19
Gender (male)		1.11 (0.93-1.33)	.24	3.69 (2.74-4.97)	<.001	–2.64 (0.36)	<.001
Parental participation (yes)		0.60 (0.37-0.97)	.04	0.78 (0.30-2.04)	.61	0.83 (0.95)	.38
Educational level (high)		0.54 (0.38-0.76)	<.001	0.57 (0.37-0.89)	.01	2.14 (0.71)	.002
Age		0.74 (0.68-0.82)	<.001	0.70 (0.62-0.78)	<.001	1.30 (0.18)	<.001
**Religion (no religion)**							
	Catholic	0.99 (0.80-1.23)	.91	0.92 (0.69-1.23)	.58	–0.03 (0.44)	.95
	Protestant	1.56 (1.08-2.25)	.02	1.95 (1.05-3.64)	.04	–1.06 (0.73)	.15
	Muslim	6.59 (4.00-10.88)	<.001	1.94 (0.93-4.05)	.08	–1.26 (0.86)	.14
	Other	1.57 (1.05-2.36)	.03	1.82 (0.95-3.48)	.07	–0.88 (0.81)	.28
Ethnicity (Dutch)		1.22 (0.88-1.71)	.25	1.50 (0.90-2.50)	.10	–0.47 (0.65)	.47
**Interaction effects**							
	Condition × gender	1.12 (0.77-1.62)	.55	0.60 (0.31-1.13)	.11	–1.00 (0.74)	.17
	Condition × educational level	1.13 (0.54-2.34)	.75	2.15 (0.91-5.10)	.08	–1.96 (1.49)	.19
	Condition × age	1.19 (0.98-1.43)	.08	1.17 (0.92-1.48)	.21	–0.32 (0.37)	.39
	Age 15 (control)	0.47 (0.24-0.91)	.03				
	Age 16 (control)	0.56 (0.30-1.05)	.07				
	Age 17 (control)	0.66 (0.34-1.28)	.22				
	Age 18 (control)	0.79 (0.38-1.63)	.52				
	Age 19 (control)	0.93 (0.40-2.17)	.87				
	High educational level			1.48 (0.38-5.74)	.57		
	Low educational level			0.46 (0.14-1.49)	.19		

^a^ Reference category of categorical variables is indicated within parentheses.

### Excessive Drinking

At the baseline assessment, 116 of 1622 (7.15%) adolescents in the experimental condition and 129 of 1027 (12.56%) adolescents in the control condition engaged in excessive drinking. At the follow-up assessment, 28 of 456 (6.1%) adolescents in the experimental and 37 of 368 (10.2%) adolescents in the control condition reported excessive drinking. There was a significant interaction effect between condition and educational level (*P*=.08). However, further analyses revealed no significant subgroup effects for either higher- or lower-educated adolescents (Table 5). Protective determinants of excessive drinking were being female (*P*<.001), a higher educational level (*P*=.01), and being younger (*P*<.001).

### Weekly Consumption

At baseline, adolescents in the experimental condition drank a mean of 3.4 (SD 8.9) standard glasses of alcohol in the previous week. Adolescents in the control condition drank a mean of 5.1 (SD 9.9) standard glasses of alcohol in the previous week. At the follow-up assessment, adolescents in the experimental condition reported a mean consumption of 3.3 (SD 7.7) and adolescents in the control condition reported a mean consumption of 4.6 (SD 8.9) standard glasses of alcohol during the previous week. Although the effects were in the expected direction, no significant effects of the intervention were found for weekly consumption. The analysis only revealed that being female (*P*<.001), having a higher educational level (*P=*.002), and being younger (*P*<.001) (all analyzed in a model without interaction terms) were significant determinants with a protective effect on weekly consumption (Table 5).

### Adherence

After the baseline assessment, adolescents in the intervention condition were supposed to start with the first game session. Of 1622 adolescents who were randomized into the experimental condition, only 1097 (67.63%) started with the first game session. Only 467 adolescents (28.79%) returned to the second and only 347 (21.39%) adolescents to the third game session. Just 27 (1.66%) adolescents returned to the fourth session at home, and zero participated in the fifth home session.

Subsequently, to investigate the effects of adherence, we decided to rerun the analyses with the subsample of the group that completed the different game sessions. We made 3 groups: the first group consisted of all adolescents who participated in the first game session, the second group consisted of adolescents who also participated in the second game session, and the third group did all 3 game sessions. Descriptive analyses of prevalence of binge drinking per age group and per adherence group can be found in the Multimedia Appendix 2. The effects for binge drinking are summarized in Table 6.

**Table 6:** Results for binge drinking for adolescents who participated in 1 or more, 2 or more, and all 3 game sessions.

Age (years)^a^	≥1 session n=1097		≥2 sessions n=467		All 3 sessions n=347	
			
	OR (95% CI)	*P*	OR (95% CI)	*P*	OR (95% CI)	*P*
Condition (control)	0.31 (0.14-0.66)	.003	0.14 (0.05-0.37)	<.001	0.13 (0.04-0.37)	<.001
Condition × age	1.34 (1.09-1.65)	.007	1.77 (1.31-2.40)	<.001	1.72 (1.23-2.40)	.002
15	0.41 (0.21-0.81)	.01	0.24 (0.11-0.57)	.001	0.22 (0.09-0.54)	.001
16	0.55 (0.29-1.04)	.07	0.43 (0.20-0.95)	.04	0.37 (0.16-0.87)	.02
17	0.74 (0.38-1.45)	.38	0.77 (0.33-1.78)	.54	0.64 (0.26-1.60)	.34
18	0.99 (0.46-2.12)	.98	1.36 (0.50-3.67)	.54	1.10 (0.37-3.27)	.86
19	1.33 (0.54-3.25)	.53	2.41 (0.73-8.02)	.15	1.89 (0.50-7.10)	.35

^a^ Reference category of categorical variables is indicated within parentheses.

Again, we found a significant interaction effect with age for all 3 groups (*P*=.007 for adolescents that participated in at least 1 session; *P*<.001 for adolescents that participated in at least 2 sessions; *P*=.002 for adolescents that engaged in all 3 game sessions). When engaging in at least 1 session, only 15-year-old adolescents reported less binge drinking (*P*=.01). The effect sizes increased when 15-year-olds adhered longer to the intervention (after 1 session: OR 0.41, 95% CI 0.21-0.81; after 2 sessions: OR 0.24, 95% CI 0.11-0.57); after 3 sessions: OR 0.22, 95% CI 0.09-0.54). A similar pattern was found for 16-year-old adolescents. There was a significant effect of the intervention after 2 sessions (OR 0.43, 95% CI 0.20-0.95, *P*=.04) which became stronger after 3 sessions (OR 0.37, 95% CI 0.16-0.87, *P*=.02). There was no significant effect for older adolescents. The analyses for excessive drinking revealed a significant interaction effect between condition and educational level (OR 2.37, 95% CI 0.98-5.73, *P*=.05) for adolescents that adhered to at least 1 session. However, the subgroup effects for higher (OR 0.87, 95% CI 0.22-3.52, *P*=.85) and lower (OR 0.46, 95% CI 0.12-1.83, *P*=.27) educated adolescents were both not significant. Weekly consumption revealed a similar result with a significant interaction effect with educational level (β=–0.22, SE 1.27, *P*=.09) for adolescents that completed at least one session, but only small and nonsignificant subgroup effects for higher- (β=–0.19, SE 0.94, *P*=.84) and lower- (β=0.17, SE 2.68, *P*=.95) educated adolescents. Furthermore, there was a significant interaction effect between condition and age on weekly consumption for adolescents that completed at least 2 sessions (β=–0.99, SE 0.52, *P*=.05) and for those who completed at least 3 sessions (β=–1.03, SE 0.59, *P*=.08); however, although the effects were more positive for the younger age groups, no effect reached a significant level. Finally, significant predictors of adherence were being Protestant, being female, being younger, having a higher educational background, and being a nonbinge drinker (Table 7).

**Table 7:** Predictors of adherence (number of sessions completed by the adolescents).

Variable^a^	β (SE)	*P*
Catholic (no religion)	0.039 (0.049)	.05
Protestant (no religion)	0.097 (0.080)	<.001
Muslim (no religion)	–0.049 (0.100)	.03
Other religion (no religion)	–0.010 (0.095)	.62
Gender (female)	–0.046 (0.040)	.02
Age	–0.138 (0.018)	<.001
Nationality (not Dutch)	0.023 (0.075)	.33
Educational level (lower)	0.088 (0.044)	<.001
Binge drinking (not binge drinking)	–0.066 (0.042)	.001

^a^ Religion was entered as a dummy variable (Catholic, Protestant, Muslim, other religion). Reference category of categorical variables is indicated within parentheses.

## Discussion

In this study, a Web-based computer-tailored intervention to reduce binge drinking in adolescents aged 15 to 19 years was tested using a cluster RCT. An overall effect of the intervention on binge drinking behavior was not found, but the intervention was effective in reducing binge drinking in adolescents aged 15 and 16 years. No additional effects were found for the secondary outcomes, excessive drinking, and weekly consumption.

That interventions to reduce alcohol use in adolescents are more effective in younger adolescents is in line with previous work [57]. Our effect sizes suggest that the intervention effect increased when adolescents adhered more to the intervention. This effect was only visible in the adolescents aged 15 and 16 years. A reason why the intervention was more successful in younger adolescents could be that younger adolescents tend to be more susceptible to peer influences than older adolescents [58]. Particularly in the second and third game sessions, we focused on social influences, such as modeling, social norms, and perceived pressure to drink from family and friends. Younger adolescents may have benefited more from this than older adolescents. Furthermore, analysis of the determinants of adherence did indicate that adolescents who adhered to the intervention were significantly younger in comparison with those who stopped prematurely. If an intervention is not used the way it is supposed to be used, its impact on health and behavior will be very limited and the public health impact probably weakened [21]. The high dropout rate of the older adolescents could explain why no effect was detected in their age group. Most adolescents initiate alcohol use between the ages of 11 to 15 years. The mean age for Dutch adolescents to first try alcohol is 13 years; the mean age for starting to drink alcohol on a weekly basis is 15 years [10]. Consequently, a possibility is that older adolescents may already have developed a kind of habit of engaging in binge drinking and other change methods more focused on changing habits, such as counterconditioning or stimulus control, are needed [59]. This might also mean that the real effect of the intervention might be stronger after a longer time period because the younger adolescents might not develop such strong habits in the next 2 years. Another possibility why older adolescents tended to drop out more could be that the game was not as appealing to those adolescents as it was to younger adolescents. Qualitative process evaluations could give more insights into what adolescents liked and what they did not like, and thereby provide future interventions with valuable input.

Adherence rates generally were low. There was a clear drop in participation between the baseline assessment and the first game session and another significant drop between the first and second game sessions. The analyses of adherence indicated that females, Protestants, younger adolescents, and nonbinge drinkers adhered better to the intervention. Particularly the last finding is not atypical in health promotion. In an intervention targeting multiple lifestyle behaviors (including alcohol use), people who adhered more to the program were also adhering more to the national health guidelines [60]. In other words, people who behaved in a more unhealthy way dropped out earlier in the program. Another study found that people with an unhealthy lifestyle were more likely to visit a health intervention website, but that people with a healthier lifestyle were more likely to complete the health intervention [61]. Yet, as health promotion programs are particularly important for groups that do not already have a healthy lifestyle, further research is definitely needed to identify how to better involve binge-drinking adolescents. Perhaps more attention needs to be directed toward premotivational determinants, such as knowledge, cues to action, and risk perception [34,62]. Starting an intervention with the focus on these factors and raising awareness that there is a problem with binge drinking might increase adolescents’ willingness to reconsider and change their behavior [63].

In our intervention, we tried to motivate adolescents to adhere to the intervention by designing a serious game that carried computer-tailored advice. Although we did not test the specific effect of the game on motivation (eg, by comparing it to a nongame intervention), adherence rates were far from optimal. A possible explanation might be that alcohol use is very common among Dutch adolescents [10,12,32] and adolescents probably do not feel the disturbing negative consequences of alcohol yet. Rather, they experience the positive aspects that come with alcohol use, such as facilitating social interaction, and they might not want to change their alcohol use [64]. Furthermore, because participation was voluntary, adolescents were aware that they could stop participation at any point without having to indicate the reasons why. This could have caused adolescents to drop out of the intervention prematurely and is a consequence of the low threshold to participate in Web-based interventions (ie, it is easy to start participating but also as easy to stop participating).

Another point was that whole schools dropped out before and during the intervention. The differences in characteristics of adolescents who did not return to the follow-up assessment compared to those who did return (adolescents who dropped out were older, male, had a lower educational background, were less likely to be religious, were more often non-Dutch, were more likely to be binge drinkers, excessive drinkers, and had a higher weekly consumption) can partly be explained by the dropout of the whole school. Furthermore, comparable characteristics of people who dropped out of the follow-up assessment have been reported in other studies as well [13,19,42].

High dropout rates in Web-based interventions are not uncommon [16,65]; therefore, a 50% dropout rate was taken into account in the power calculation. However, dropout rates at follow-up were higher than the expected 50%, which could also result in too little power of the analyses to detect possible effects of this intervention [21]. Although we sent emails to remind adolescents to return to the intervention website, there might be other possibilities to increase revisiting numbers. Newer research is focusing on the content and timing [66] of those reminders and how other prompts, such as a text message to a mobile phone, can remind participants to revisit the intervention website [67].

Also important is our finding that parental participation in the parental component was associated with significantly lower rates of binge drinking among adolescents, which might be an indication that the parental component was an important addition to the intervention. However, due to methodological choices in parent recruitment (ie, adolescents invited their parents to participate), those data are observational rather than experimental and strong claims about the effect cannot be made. It is possible that other factors, such as family attachment, influenced the positive results. However, the low participation of parents is notable. On the one hand, just a small proportion of adolescents actually invited their parents to take part in the intervention. That could be an indication that adolescents do not feel the need or do not want to talk about the subject with their parents. On the other hand, of the 199 adolescents who invited their parents, which is likely a very selective group of adolescents already, only 91 parents actually visited the website. Other studies that focused on parent-child communication about risky sexual behavior also reported low attendance rates of parents [68,69]. Generally, interest in Internet-delivered interventions has been shown to be quite low [16,70]. It could also indicate that parents may not feel involved in the alcohol use of their child. This has also come to surface in focus group interviews held with adolescents and parents [32], where parents indicated that they stopped talking with their child about alcohol and stopped setting rules when they turned age 16. Even after the change in law, there seems to be no immediate change in this parental behavior.

A strength of this study is that it is theory-based and was preceded by extensive qualitative and quantitative research. Furthermore, the target group was included and consulted during the whole development process [32]. However, despite all these efforts to make the intervention as interesting and appealing to the target group as possible, the dropout rates were very high, which made it very difficult to reveal effects of the intervention. Further, although some significant effects on behavior were found, these effects have to be interpreted with caution because of the high dropout rate.

In this study, only relatively short-term outcomes of the intervention were assessed. It is advisable to add more long-term assessments to evaluate what the true effects are after 12 or 24 months, or even after a longer time period.

Another limitation is that all outcome measures were based on self-reports, which is more likely to result in greater underestimation of alcohol use compared to daily diaries [71]. This underestimation is probably mostly caused by forgetting [48]. However, we tried to keep self-reports as accurate as possible (eg, by asking for alcohol use in the previous week and not in a typical week). Furthermore, because the groups were randomized, this underestimation is probably equally distributed among the intervention and control groups and, therefore, does not influence the overall results of the study.

Finally, adolescents from the experimental and control conditions differed on alcohol use (ie, binge drinking, excessive drinking, and weekly consumption) as well as on several baseline characteristics (ie, gender, age, educational background, religion) which was probably caused by the relatively high dropout of schools in the control condition after randomization (5 schools withdrew participation before the baseline assessment). There were no differences on baseline drinking measures for the returning sample, but they were added in the analyses as covariates to control for the baseline differences of the whole sample.

Computer-tailored feedback can be an effective way to reduce binge drinking in adolescents aged 15 and 16 years. Also, participation of parents in those interventions may be beneficial and more research is needed to increase parental involvement. Further research is needed to increase adherence to eHealth interventions and to implement these interventions in practice; thereby, increasing their effectiveness and public health impact.

## Appendix

Multimedia Appendix 1 CONSORT-eHealth Checklist.

Multimedia Appendix 2 Prevalence rates of binge drinking per age and adherence group.

## Abbreviations

RCT: randomized controlled trial

## References

[1] Best D, Manning V, Gossop M, Gross S, Strang J (2006). Excessive drinking and other problem behaviours among 14-16 year old schoolchildren. Addict Behav.

[2] Anderson P, Baumberg B (2006). Alcohol in Europe: A Public Health Perspective.

[3] Swahn MH, Simon TR, Hammig BJ, Guerrero JL (2004). Alcohol-consumption behaviors and risk for physical fighting and injuries among adolescent drinkers. Addict Behav.

[4] Miller JW, Naimi TS, Brewer RD, Jones SE (2007). Binge drinking and associated health risk behaviors among high school students. Pediatrics.

[5] Stolle M, Sack P, Thomasius R (2009). Binge drinking in childhood and adolescence: epidemiology, consequences, and interventions. Dtsch Arztebl Int.

[6] Testa M, Livingston JA (2009). Alcohol consumption and women's vulnerability to sexual victimization: can reducing women's drinking prevent rape?. Subst Use Misuse.

[7] Bava S, Tapert SF (2010). Adolescent brain development and the risk for alcohol and other drug problems. Neuropsychol Rev.

[8] Clark DB, Thatcher DL, Tapert SF (2008). Alcohol, psychological dysregulation, and adolescent brain development. Alcohol Clin Exp Res.

[9] Zeigler DW, Wang CC, Yoast RA, Dickinson BD, McCaffree MA, Robinowitz CB, Sterling ML, Council on Scientific Affairs‚ American Medical Association (2005). The neurocognitive effects of alcohol on adolescents and college students. Prev Med.

[10] Verdurmen J, Monshouwer K, Dorsselaer S, Lokman S, Vermeulen-Smit E, Vollebergh W (2011). Jeugd en riskant gedrag.

[11] Hibell B, Guttormsson U, Ahlstrom T, Balakireva O, Bjarnason T, Kokkevi K (2009). The 2007 ESPAD Report: Substance Use Amongst Students in 35 European Countries.

[12] De Looze LM, Van Dorsselaer DS, De Roos RS, Verdurmen J, Stevens G, Gommans R (2014). HBSC 2013 Gezondheid, welzijn en opvoeding van jongeren in Nederland.

[13] Schulz DN, Candel MJ, Kremers SP, Reinwand DA, Jander A, de VH (2013). Effects of a Web-based tailored intervention to reduce alcohol consumption in adults: randomized controlled trial. J Med Internet Res.

[14] (2013). Centraal Bureau voor de Statistiek.

[15] de Vries H, Brug J (1999). Computer-tailored interventions motivating people to adopt health promoting behaviours: introduction to a new approach. Patient Educ Couns.

[16] Kohl LF, Crutzen R, de Vries NK (2013). Online prevention aimed at lifestyle behaviors: a systematic review of reviews. J Med Internet Res.

[17] Krebs P, Prochaska JO, Rossi JS (2010). A meta-analysis of computer-tailored interventions for health behavior change. Prev Med.

[18] Lustria ML, Noar SM, Cortese J, Van Stee SK, Glueckauf RL, Lee J (2013). A meta-analysis of web-delivered tailored health behavior change interventions. J Health Commun.

[19] Elfeddali I, Bolman C, Candel MJ, Wiers RW, de Vries H (2012). Preventing smoking relapse via Web-based computer-tailored feedback: a randomized controlled trial. J Med Internet Res.

[20] Kelders SM, Kok RN, Ossebaard HC, Van Gemert-Pijnen JE (2012). Persuasive system design does matter: a systematic review of adherence to web-based interventions. J Med Internet Res.

[21] Eysenbach G (2005). The law of attrition. J Med Internet Res.

[22] Pate RR, Saunders RP, Ward DS, Felton G, Trost SG, Dowda M (2003). Evaluation of a community-based intervention to promote physical activity in youth: lessons from Active Winners. Am J Health Promot.

[23] Crutzen R, de Nooijer J, Brouwer W, Oenema A, Brug J, de Vries NK (2011). Strategies to facilitate exposure to internet-delivered health behavior change interventions aimed at adolescents or young adults: a systematic review. Health Educ Behav.

[24] Connolly TM, Boyle EA, MacArthur E, Hainey T, Boyle JM (2012). A systematic literature review of empirical evidence on computer games and serious games. Comput Educ.

[25] DeSmet A, Van Ryckeghem RD, Compernolle S, Baranowski T, Thompson D, Crombez G, Poels K, Van Lippevelde W, Bastiaensens S, Van Cleemput K, Vandebosch H, De Bourdeaudhuij I (2014). A meta-analysis of serious digital games for healthy lifestyle promotion. Prev Med.

[26] Papastergiou M (2009). Digital game-based learning in high school computer science education: impact on educational effectiveness and student motivation. Comput Educ.

[27] Tüzün H, Yılmaz-Soylu M, Karakuş T, İnal Y, Kızılkaya G (2009). The effects of computer games on primary school students’ achievement and motivation in geography learning. Comput Educ.

[28] van der Vorst H, Engels RC, Meeus W, Deković M, Van Leeuwe J (2005). The role of alcohol-specific socialization in adolescents' drinking behaviour. Addiction.

[29] van der Vorst H, Engels RC, Meeus W, Deković M (2006). The impact of alcohol-specific rules, parental norms about early drinking and parental alcohol use on adolescents' drinking behavior. J Child Psychol Psychiatry.

[30] Spijkerman R, van den Eijnden RJ, Huiberts A (2008). Socioeconomic differences in alcohol-specific parenting practices and adolescents' drinking patterns. Eur Addict Res.

[31] Turrisi R, Jaccard J, Taki R, Dunnam H, Grimes J (2001). Examination of the short-term efficacy of a parent intervention to reduce college student drinking tendencies. Psychol Addict Behav.

[32] Jander A, Mercken L, Crutzen R, de Vries H (2013). Determinants of binge drinking in a permissive environment: focus group interviews with Dutch adolescents and parents. BMC Public Health.

[33] (2014). Rijksoverheid.

[34] de Vries H, Mudde A, Leijs I, Charlton A, Vartiainen E, Buijs G, Clemente MP, Storm H, González NA, Nebot M, Prins T, Kremers S (2003). The European Smoking Prevention Framework Approach (EFSA): an example of integral prevention. Health Educ Res.

[35] De Vries H, Mudde A (1998). Predicting stage transitions for smoking cessation applying the attitude-social influence-efficacy model. Psychol Health.

[36] Fishbein M (1980). A theory of reasoned action: some applications and implications. Nebr Symp Motiv.

[37] Ajzen I (1991). The theory of planned behavior. Organ Behav Hum Dec.

[38] Bandura A (1986). Social Foundations of Thought and Action: A Social Cognitive Theory.

[39] Janz NK, Becker MH (1984). The health belief model: a decade later. Health Educ Behav.

[40] Weinstein ND (1988). The precaution adoption process. Health Psychol.

[41] Prochaska JO, DiClemente CC, Norcross JC (1992). In search of how people change. Applications to addictive behaviors. Am Psychol.

[42] Schulz DN, Kremers SP, Vandelanotte C, van Adrichem MJ, Schneider F, Candel MJ, de Vries H (2014). Effects of a web-based tailored multiple-lifestyle intervention for adults: a two-year randomized controlled trial comparing sequential and simultaneous delivery modes. J Med Internet Res.

[43] Stanczyk N, Bolman C, van Adrichem M, Candel M, Muris J, de Vries H (2014). Comparison of text and video computer-tailored interventions for smoking cessation: randomized controlled trial. J Med Internet Res.

[44] de Vries H, Backbier E (1994). Self-efficacy as an important determinant of quitting among pregnant women who smoke: the phi-pattern. Prev Med.

[45] Dijkstra A, De Vries H (1999). The development of computer-generated tailored interventions. Patient Educ Couns.

[46] Jander A, Crutzen R, Mercken L, de Vries H (2014). A Web-based computer-tailored game to reduce binge drinking among 16 to 18 year old Dutch adolescents: development and study protocol. BMC Public Health.

[47] Wechsler H, Dowdall GW, Davenport A, Rimm EB (1995). A gender-specific measure of binge drinking among college students. Am J Public Health.

[48] Lemmens P, Tan ES, Knibbe RA (1992). Measuring quantity and frequency of drinking in a general population survey: a comparison of five indices. J Stud Alcohol.

[49] Kaiser HF (1960). The application of electronic computers to factor analysis. Educ Psychol Meas.

[50] Dunn TJ, Baguley T, Brunsden V (2014). From alpha to omega: a practical solution to the pervasive problem of internal consistency estimation. Br J Psychol.

[51] Peters GJY (2014). The alpha and the omega of scale reliability and validity: Why and how to abandon Conbach's alpha and the route towards more comprehensive assessment od scale quality. Euro Health Psychologist.

[52] Spybrook J, Bloom H, Congdon R, Hill C, Martinez A, Raudenbush S (2011). Optimal Design Plus Empirical Evidence: Documentation for the “Optimal Design” Software.

[53] Candel MJ, Van Breukelen GJ (2010). Sample size adjustments for varying cluster sizes in cluster randomized trials with binary outcomes analyzed with second-order PQL mixed logistic regression. Stat Med.

[54] Molenberghs G, Kenward M (2007). Missing data in clinical studies.

[55] Hayes AF, Matthes J (2009). Computational procedures for probing interactions in OLS and logistic regression: SPSS and SAS implementations. Behav Res Methods.

[56] Rosenthal JA (1996). Qualitative descriptors of strength of association and effect size. J Soc Serv Res.

[57] Perry CL, Williams CL, Komro KA, Veblen-Mortenson S, Stigler MH, Munson KA, Farbakhsh K, Jones RM, Forster JL (2002). Project Northland: long-term outcomes of community action to reduce adolescent alcohol use. Health Educ Res.

[58] Crockett L, Petersen A, Millstein SG, Petersen AC, Nightingale EO (1993). Adolescent development: health risks and opportunities for health promotion. Promoting the Health of Adolescents.

[59] Bartholomew L, Parcel G, Kok G, Gottlieb N, Fernandez M (2011). Planning Health Promotion Programs: An Intervention Mapping Approach. third ed.

[60] Schulz DN, Schneider F, de Vries H, van Osch LA, van Nierop PW, Kremers SP (2012). Program completion of a web-based tailored lifestyle intervention for adults: differences between a sequential and a simultaneous approach. J Med Internet Res.

[61] Schneider F, van Osch L, Schulz DN, Kremers SP, de Vries H (2012). The influence of user characteristics and a periodic email prompt on exposure to an internet-delivered computer-tailored lifestyle program. J Med Internet Res.

[62] de Vries H, Kremers SP, Smeets T, Brug J, Eijmael K (2008). The effectiveness of tailored feedback and action plans in an intervention addressing multiple health behaviors. Am J Health Promot.

[63] Prochaska JO, Redding CA, Evers KE, Glanz BKR BK, Viswanath K (2008). The Transtheoretical Model and stages of change. Health Behavior and Health Education: Theory, Research, and Practice.

[64] Kuntsche E, Knibbe R, Gmel G, Engels R (2005). Why do young people drink? A review of drinking motives. Clin Psychol Rev.

[65] de Vries H, Logister M, Krekels G, Klaasse F, Servranckx V, van Osch L (2012). Internet based computer tailored feedback on sunscreen use. J Med Internet Res.

[66] Schneider F, de Vries H, Candel M, van de Kar A, van Osch L (2013). Periodic email prompts to re-use an internet-delivered computer-tailored lifestyle program: influence of prompt content and timing. J Med Internet Res.

[67] Cremers H, Mercken L, Crutzen R, Willems P, de Vries H, Oenema A (2014). Do email and mobile phone prompts stimulate primary school children to reuse an Internet-delivered smoking prevention intervention?. J Med Internet Res.

[68] DiIorio C, McCarty F, Resnicow K, Lehr S, Denzmore P (2007). REAL men: a group-randomized trial of an HIV prevention intervention for adolescent boys. Am J Public Health.

[69] Anderson NL, Koniak-Griffin D, Keenan CK, Uman G, Duggal BR, Casey C (1999). Evaluating the outcomes of parent-child family life education. Sch Inq Nurs Pract.

[70] Bennett GG, Glasgow RE (2009). The delivery of public health interventions via the Internet: actualizing their potential. Annu Rev Public Health.

[71] Sobell LC, Cellucci T, Nirenberg TD, Sobell MB (1982). Do quantity-frequency data underestimate drinking-related health risks?. Am J Public Health.

