# Supplementary material for: Effects of a Web-Based Computer-Tailored Game to Reduce Binge Drinking Among Dutch Adolescents: A Cluster Randomized Controlled Trial
Source: J Med Internet Res. 2016 Feb 3;18(2):e29. doi: 10.2196/jmir.4708 (PMC4757780; doi:10.2196/jmir.4708)
Supplement: Multimedia Appendix 2 [file jmir_v18i2e29_app2.pdf]

Multimedia appendix 1. Prevalence rates binge drinking per age and adherence group.

| Age (years) | ≥ 1 session, n/n (%) | | ≥ 2 sessions, n/n (%) | | ≥ 3 sessions, n/n (%) | |
|---|---|---|---|---|---|---|
| | Baseline | Follow-up | Baseline | Follow-up | Baseline | Follow-up |
| | n=1093 | n=352 | n=465 | n=191 | n=345 | n=140 |
| 15 | 125/471 (26.5) | 41/140 (29.3) | 62/225 (27.6) | 21/84 (25) | 44/169 (26.0) | 14/67 (21) |
| 16 | 158/336 (47.2) | 52/120 (43.3) | 70/141 (49.6) | 32/72 (44) | 47/101 (46.5) | 21/48 (44) |
| 17 | 90/164 (54.9) | 31/50 (63) | 36/54 (66) | 14/20 (70) | 27/42 (64) | 11/17 (65) |
| 18 | 57/76 (75) | 11/19 (57) | 23/27 (85) | 4/7 (57) | 15/19 (79) | 0/2 (0) |
| 19 | 33/46 (73) | 14/23 (61) | 16/18 (89) | 7/8 (87) | 12/14 (85) | 6/6 (100) |
| total | 463/1093 (42.36) | 149/352 (42.5) | 207/465 (44.5) | 78/191 (40.8) | 145/345 (42.0) | 52/140 (37.1) |
